# Current state of orthodontic patients under Bisphosphonate therapy

**DOI:** 10.1186/1746-160X-9-10

**Published:** 2013-04-04

**Authors:** Elena Krieger, Collin Jacobs, Christian Walter, Heinrich Wehrbein

**Affiliations:** 1 Department of Orthodontics, Medical Centre of the Johannes-Gutenberg-University Mainz, Augustusplatz 2, 55131, Mainz, Germany; 2 Department of Cranio-Maxillo-Facial Surgery, Medical Centre of the Johannes Gutenberg-University Mainz, Augustusplatz 2, 55131, Mainz, Germany

**Keywords:** Medication, Side effects, Bone metabolism, Orthodontic treatment, Tooth movement

## Abstract

**Background:**

Bisphosphonates are a common medication for the prevention and therapy of osteoporosis, but are also applied for tumor diseases. They affect bone metabolism, and therefore also orthodontic treatments, but how it does has yet not been definitively clarified. Therefore, the aim of this research was to evaluate and demonstrate the reported effects and the current state of scientific research regarding orthodontic treatment and bisphosphonate medication exclusively in humans.

**Material and methods:**

A systematic research of the literature for selected keywords in the Medline database (Pubmed) as well as a manual search was conducted. The following search terms were used: ‘Bisphosphonate’ in combination with: orthodontic, orthodontic treatment, tooth movement.

**Findings:**

To date, only nine reported patients (case reports/series) and one original article (retrospective cohort study) regarding orthodontic treatment under bisphosphonate medication in humans have been published. Decelerated tooth movement with increased side effects (especially in high-risk patients) and longer treatment duration was reported in some articles. Patients with initial spacing or extraction cases had a higher risk of incomplete space closure and poor root parallelism.

**Conclusions:**

Orthodontic tooth movement under bisphosphonate medication is possible, especially in low-risk patients (low dose and short period of intake). But the treatment is still not predictable, especially in high-risk patients. Therefore, the altered bone metabolism and higher extent of side effects should be considered in treatment planning, especially in extraction cases or high-risk patients. Regardless, longer treatment duration, decelerated tooth movement, and more side effects, e.g., incomplete space closure and poor root parallelism, should be expected, especially in extraction cases or space closure.

## Introduction

Bisphosphonates (BPs) are used in patients with metabolic bone diseases such as osteoporosis and in patients with malignomas and metastases to the bone. They have an inhibiting effect on osteoclastic activity and therefore decrease the bone resorption. The half-life can be more than 10 years [1]. One side effect is a bisphosphonate associated osteonecrosis of the jaws (BP-ONJ) [2-8]. The risk of BP-ONJ seems to depend on the type of BP, dose, duration and application form (intravenous or oral). Patients with IV BP and malignant disease are at a higher risk compared to patients with oral BP and benign diseases [2-8].

To date, the altered bone metabolism and the following consequences to orthodontic therapy and, possibly, revised indications have not been clarified. Only statements regarding dentosurgical therapies have been published by the field societies [5-7]. Concerning orthodontic aspects, there are no guiding principles.

Therefore, the aim of this study was to analyze the literature reporting on the combination of orthodontic treatment and BP medication exclusively in humans.

## Material and methods

A systematic research in PubMed was performed covering publications from Jan 2000- Jan 2013. In conjunction with this, a manual search of national professional orthodontic journals published in German but not listed in PubMed was conducted. In addition, the citations in identified articles were analyzed as well. Inclusion criteria were reports on humans, BP treatment, and orthodontic treatment. Exclusion criteria were reports on animals, and a lack of BP and/or orthodontic treatment. The following search terms were used:

‘Bisphosphonate’ in combination with: orthodontic, orthodontic treatment, tooth movement.

## Results

Altogether, without redundancies, 28 articles were found. Only seven articles met the inclusion criteria: four case reports [9-12], two case series with two [13] and three patients respectively [14], and one original article with 20 BP medicated patients [15].

The original article presented a retrospective cohort study with women <50 years-of-age [15]. The study was approved by the institutional review board at the University of Washington in Seattle. Orthodontists from the USA were invited to perform case reviews of their patients regarding women <50 years-of-age. The sample comprised 113 female patients, divided into two groups, one with (n = 20; 19 with oral BP, 1 with IV BP) and one without (n = 93) BP treatment. BP patients that were treated orthodontically with extractions and space closing had to be treated significantly longer, and they had a higher risk of incomplete space closure and poor root parallelism at the end of treatment. No BP-ONJ was described. Both groups had an alignment of the incisors of less than 1 mm discrepancy.

The following is a summary of the nine reported patients on BP with orthodontic treatment (Table 1) [9-14]:

1. A 35-year-old middle to high-risk female patient on oral alendronate and corticosteroids due to Addison’s disease underwent orthodontic treatment lasting 30 months. Orthodontic space closing and root paralyzing was described as difficult and tooth movement decelerated. An orthopantomogram at the end of the treatment showed radiopaque areas, sclerotic lines, and denser bone. Widened periodontal ligaments (PDL) were only found at the extraction site. Further orthopantomograms 20 months after treatment and 12 months after discontinuing BP treatment revealed no more sclerotic areas of bone and no apical root resorption [13].

2. A 77-year-old high-risk male patient with sacral plasmocytoma with radiation, chemotherapy, and IV BP (zoledronate) had orthodontic space closure after extraction of the lingually-positioned tooth 42 (residual gap of 2 mm). After developing multiple myeloma, BP-ONJ developed in the right mandible, and orthodontic treatment was stopped at month 13. Instead of bodily movement, only tipping of the crowns had occurred and the tooth movement was slowed [13].

3. A 60-year-old low-risk female patient (oral alendronate for 18 months) with osteoporosis had a severe right posterior open bite which was scheduled to be closed orthodontically. Before starting the treatment, a mild sclerosis was observed at tooth 47. Two years later sclerotic bone occurred around the treated teeth with a widened PDL. BP was stopped after 3.5 years in total. The radiographic signs were enhanced and tooth movement was decelerated so that orthodontic treatment was discontinued after 4.5 years. No apical root resorption occurred [14].

4. A 50-year-old low-risk female patient underwent space closing after periodontal treatment and extraction of tooth 34. Alendronate intake due to osteoporosis was not known by the orthodontist. BP treatment was stopped after 12 months because of esophagitis (common side effect of BP). In 19 months of treatment, only crown-tipping occurred instead of bodily movement, and tooth movement was slowed down. Hypermineralized areas were found at the extraction site. Even 13 months after stopping BP and completion of further orthodontic treatment, extensive mobility and widened PDL were observed [14].

5. A 74-year-old low-risk female patient with 36 months of oral alendronate use (for osteoporosis) and severe bone loss because of periodontal disease had tooth 31 extracted with orthodontic space closure. BP treatment was stopped 3 months prior to orthodontic treatment, which took 14 months. On initial radiographs mild sclerosis around the mandibular molars and obscured anterior PDL gaps were diagnosed. The final radiographs showed mild sclerotic and PDL spaces, mild apical root resorption of mandibular incisor, and no increased mobility. Space closure and alignment were considered successful [14].

6. A 68-year-old low-risk female patient taking oral ibandronate for osteoporosis had an augmentation of the right sinus with autologous particulated bone from the anterior mandible. For skeletal anchorage a rigid fixation plate was inserted at the os zygomaticum. After 6 months of healing, teeth 13, 14, and 15 were distalized using the bone anchor, and all teeth were aligned (duration: 26 weeks). After distalization, all teeth were orthodontically aligned and dental implants inserted in the posterior right maxilla. The overall duration of orthodontic treatment was not specified, and no complications related to the BP medication were observed [9].

7. “Bloodless tooth extraction” after orthodontic extrusion was performed in a 70-year-old low-risk female patient with long-term oral BP therapy for osteoporosis. The first and second right mandibular molars were hemisected. The distal roots were orthodontically extruded for several weeks and afterwards extracted [10].

8. An alveolar bone grafting was performed on a 15-year-old high-risk female patient with polyostotic fibrous dysplasia as well as a surgically-treated cleft lip and palate. BP treatment started at the age of 13 with 4 times IV pamidronate. Teeth 12 and 22 were missing, with an anterior crowding in the mandible and an anterior cross bite. It took 6 months after the graft surgery, until an obvious bone bridge at the recipient site became visible and orthodontic treatment was begun (sign of decreased healing potential of bone). Therefore, the orthodontic treatment was performed over a longer time and less aggressive. After 3.5 years of orthodontic treatment, the patient had a sufficient occlusion [11].

9. Dental implants were inserted in the lateral segments of a 66-year-old low-risk female patient and used as skeletal anchorage during orthodontic treatment. Upper and lower front teeth were in- and retruded (duration: 13 months). BP medication for osteoporosis (alendronate oral) by her general practitioner was begun without knowledge of the dental facility and stopped after 7 months because of recurrent colitis (common side effect). After treatment, the patient had enlarged PDL, sclerotic bone areas, apical root resorptions of the upper frontal teeth, and increased mobility of the mandibular incisors [12].

**Table 1:** **Presentation of the nine reported cases of orthodontically treated patients under BP medication [**[9][14]**]**

	**Case 1 [**[13]**]**	**Case 2 [**[13]**]**	**Case 3 [**[14]**]**	**Case 4 [**[14]**]**	**Case 5 [**[14]**]**	**Case 6 [**[9]**]**	**Case 7 [**[10]**]**	**Case 8 [**[11]**]**	**Case 9 [**[12]**]**
**Anamnesis**	Addison´s disease (primary adrenal insufficiency)	Sacral plasmacytoma	Osteoporosis prevention	Osteoporosis prevention	Osteoporosis prevention	Osteoporosis prevention	Osteoporosis prevention	Polyostotic fibrous dysplasia, bilateral cleft lip and palate	Osteoporosis prevention
**Age, gender**	35, female	77, male	60, female	50, female	74, female	68, female	70, female	15, female	66, female
**Medication (dose rate)**	1 / week Alendronate 70 mg oral; 1 / day hydrocortisone 30 mg, 1 / day fludrocortisone acetate 0.10 mg, 1 / day calcium with vitamin K + D 1000–1500 mg	1 / month Zolendronate 500 mg iv; further medication (chemotherapy)	Alendronate oral, dose not specified	Alendronate oral, dose not specified	Alendronate oral, dose not specified; drug holiday 3 months before beginning and during orthodontic treatment	Ibandronate oral, dose not specified	oral, not further specified	4 cycles of Pamidronate intravenous, 90 or 135 mg every 5 months (45 mg/day, over 2 to 3 days)	1 / week Alendronate 70 mg oral
**High/low risk patient**	medium high risk (due to the corticosteroid medication)	high risk	low risk	low risk	low risk	low risk	low risk	high risk	low risk
**Intake of BP during orthodontic treatment?**	yes, 30 months	yes	yes, 24 months	yes	no	yes	yes	no	yes, 6 months
**How long intake before orthodontic treatment and all together?**	41 months before, 58 months all together	11 months before	18 months before, 42 months all together	6 months before, 12 months all together	36 months before	not specified	10 years before	2 years before	1 month before, 7 months all together
**Intake of BP known by the orthodontists?**	yes	yes	yes	no	yes	yes	yes	yes	no, emerged during treatment
**Orthodontic treatment plan**	unilateral space closure after extraction of tooth 14 and 44	space closure after extraction of tooth 42	closing of right posterior open bite	space closure after extraction of tooth 34	space closure after extraction of tooth 31	alignment, distalization 13–15 with skeletal anchorage	orthodontic extrusion of distal root of teeth 36,37	bone graft for the alveolar cleft to align the upper canines, providing anterior crossbite	intrusion/retrusion of upper and lower frontal teeth using skeletal anchorage
**Duration of orthodontic treatment (months)**	30	13 (abortion)	54 (abortion)	19	14	65 for distalization, in total not specified	7 respectively 5 weeks	42	11
**Radiographs (end of orthodontic treatment)**	radiopaque areas, sclerotic lines, denser bone and widened periodontal ligament on extraction site in the mandible	osteonecrosis in the right mandible (apical 44, 45 and dental implant in region 46)	sclerotic bone areas, widened periodontal gaps	at the extraction site hyper-mineralized areas, sclerotic bone, widened periodontal gaps	mandibular incisors: mild sclerosis and periodontal spaces, mild root resorptions	no abnormality	bone apposition in the apical area	not specified	sclerotic bone areas, widened periodontal gaps, mild apical root resorption of maxillary incisors
**Apical root resorption**	none	not specified	none	not specified	yes	not specified	not specified	not specified	yes
**Tooth movement**	decelerated	decelerated	decelerated	decelerated	not decelerated	not specified	not specified	not specified	not decelerated
**Complications /noticeable problems**	closing and paralyzing of the roots	osteonecrosis with ulceration; no bodily movement, only tipping of the crowns	despite stopping medication, side effects enhanced	compromised parallel roots; mandibular incisor mobility	no increased mobility	no complications reliable to the medication of BP	no clinical evidence of inflammation or pain, and the radiograph	not specified	higher mobility of the frontal lower teeth

## Discussion

The number of adult and elderly patients requiring orthodontic treatment is increasing, leading to new challenges for the provider: fundamental differences in anatomical and physiological conditions, altered periodontal situations, and different medical histories with different medications, all of which have a considerable influence on orthodontic planning and treatment [16]. In addition to the anamnesis, good communication between the dentist/orthodontist and the general practitioner is necessary. Two of the nine reported patients were medicated with a BP without orthodontist’s knowledge [12,14].

The medical history must be investigated far into the past due to the long half-life of several medications [13,14]. Drug holidays are controversially discussed: the long half-life of BPs in the bone (10–12 years) [1,14] versus the effects of the BPs on the soft tissue including the vascular system [17].

Known risk factors for BP-ONJ development include the specific characteristics of the BP taken (type, dose, duration of use, and administration route) as well as further medications such as steroids taken in parallel [2-7]. In the analyzed articles BP-ONJ only occurred in one high-risk patient suffering from multiple myeloma who was treated with IV zoledronate and chemotherapy [13].

The analyzed cases show that the high-risk patient had the most severe side effects, but that even low-risk patients have some. The cohort study reported that low-risk BP-patients with extractions or initial spacing had higher odds of incomplete space closure, poor root parallelism and longer treatment duration [15]. The case reports/series with extractions reported on compromised root paralleling and crown-tipping instead of bodily movement [13,14].

Radiographic signs such as sclerosis of the alveolar bone and widened PDL were found [12-14]. Apical root resorptions in the anterior region were described in two of the nine patients [12,14], while none were described in three cases [13,14] and were not specified in four other cases [9-11,14].

The data regarding root resorption under BP influence are not consistent. One animal experimental study has observed root resorptions [18], while others have found inhibiting effects of BP on root resorption [19-23].

Regarding the common use of BP for osteoporosis in postmenopausal women, an untreated control group of rats was compared with a group of ovariectomized rats and a group of ovariectomized rats with zolendronate treatment [24]. The ovariectomized/zoledronate group showed similar results as the control group. The ovariectomized group had the highest amount of root resorptions and tooth movement [24].

Regarding the velocity of tooth movement, some of the described patients had decreased movement [13-15], but others did not [12,14] or were not specified [9-11]. Several animal experimental studies have reported slowed tooth movement, partly in a dose-depending manner [19-23,25].

## Conclusion

Due to the increasing number of older patients requiring orthodontic treatment, the number of patients with a medical history including BP treatment is increasing. Regarding the half-life of BPs, an intensive examination of the medical history before treatment should be obligatory. Due to the missing scientific evidence, orthodontic tooth movement in patients exposed to BPs is still unpredictable, and risk stratification should be performed. The altered bone metabolism and higher extent of side effects should be considered in treatment planning, especially in extraction cases or high-risk patients. Also, longer treatment duration, decelerated tooth movement and, in patients with initial spacing or extraction cases, incomplete space closure and poor root parallelism should be expected.

## Competing interest

The authors declare that they have no competing interest.

## Authors’ contributions

EK carried out the conception and design of the study. She assembled the data, conducted the analysis and interpretation of data, and drafted the manuscript. CJ is a specialist in the field Bisphosphonates and orthodontics. He was involved in conception and design of the study, also in analysis and interpretation of data, and drafting the manuscript. As a Cranio-Maxillo-Facial Surgeon and specialist in the field Bisphosphonates, CW had an indispensable contribution by conceiving and participate the design of the study, and also helped to draft the manuscript. HW, Head of the Department, conceived of the study, and participated in its design and coordination and helped to draft the manuscript. All authors read and approved the final manuscript.
